# Damage Evolution Constitutive Behavior of Rock in Thermo-Mechanical Coupling Processes

**DOI:** 10.3390/ma14247840

**Published:** 2021-12-18

**Authors:** Suran Wang, Haohao Liao, Youliang Chen, Tomás Manuel Fernández-Steeger, Xi Du, Min Xiong, Shaoming Liao

**Affiliations:** 1 Department of Geotechnical Engineering, College of Civil Engineering, Tongji University, 1239 Siping Road, Shanghai 200092, China; wangsuran@tongji.edu.cn (S.W.); 1310298@tongji.edu.cn (M.X.); engcent@tongji.edu.cn (S.L.); 2 Department of Civil Engineering, University of Shanghai for Science and Technology, 516 Jungong Road, Shanghai 200093, China; chenyouliang2001@163.com (Y.C.); duxijl@163.com (X.D.); 3 Institut für Angewandte Geowissenschaften, Technische Universität Berlin, Ernst-Reuter-Platz 1, BH 3-1, 10587 Berlin, Germany; Fernandez-steeger@tu-berlin.de

**Keywords:** rock mechanics, unified constitutive model, damage evolution, coupling effect, thermo-load rock

## Abstract

For thermal and loaded rock in engineering structures for some projects, triple-shear Drucker–Prager yield criteria, compaction coefficient *K*, damage variable correction factor *δ*, and thermal damage variable *D_T_* are introduced in a new thermomechanical (TM) constitutive model for the entire process. The compaction stage of rock in uniaxial compression test and the strain softening of rock caused by thermal attack are considered in this article. The damage evolution of rocks is described by a damage variable and a constitutive equation, which are in agreement with the actual thermal experimental breakage. The uniaxial compressive strength of granite subjected to a TM coupling effect can be predicted properly by this new unified constitutive model. The new TM unified constitutive model considering the compaction stage and post-failure stage is in good agreement with the test curves throughout the entire process. The coupling effect of heat and load in the total damage of rock has obvious nonlinear properties, but the coupling effect significantly weakens the specimens. By using the new TM unified constitutive model, the whole process of changes in rock damage with strain after high temperature can be calculated. Meanwhile, the model well represents the stress–strain curve at the post-failure stage. It is expected that this model can provide references for studying the mechanical response of the rock damage propagation characteristics in the future.

## 1. Introduction

With the increasing number of projects involving mining, cracked reservoir extraction, and underground nuclear waste disposal, there have been concerns about the stability of rocks under complex conditions, such as in a thermomechanical environment. There are certain situations of rock breakage under the coupling effect of heat and load exemplified all over the world. Research on the thermo-load breakage of some selected rocks from countries around the world was conducted by Sygala et al. [1]. The mechanical properties of rocks after heat treatment have also been analyzed; the results indicate that the physical and mechanical properties of the rocks are affected by porosity, density, and mineral composition (Rao et al., 2008; Zhang et al., 2015; Roy and Singh, 2016; Liang et al., 2006; Chen et al., 2017) [2,3,4,5,6]. Researchers observed and analyzed the failure behavior and mechanical properties of Pingdingshan sandstone from room temperature up to 300 °C via laboratory experiments; the results show that the tensile strength increased with temperatures from 25–150 °C (Zuo et al., 2012) [7]. However, it should be noted that the results with effective confining pressure would be more accurate. The thermal expansion of three water-saturated rocks with effective confining pressures at high temperatures was measured. The thermal expansion at confining pressure had an increasing effect on strength, elastic moduli, sound velocity, thermal conductivity, and porosity (Stephen and John, 1983) [8].

The Brazilian disc tests and the three-point bending tests were carried out, and the composition and the structure of minerals were regarded as the greatest influences on the mechanical properties of rocks (Rao et al., 2007) [9]. In addition, 13 samples from diverse locations in Morocco were collected and thermally cycled between 20 and 650 °C; the results proved that limestone, marble, and granite cannot withstand thermal cycling, and their hardness decreased after each cycle, while quartz and calcite in sandstone were the principal minerals controlling the physical properties of the rock (Tiskatine et al., 2016) [10]. To characterize the changes in the mineralogy and microstructural texture of two sedimentary rocks, samples were subjected to temperatures up to 1200 °C; the results showed that the unconfined compressive strength of both rock types tended to increase when the temperature increased up to 900 °C, beyond which the unconfined compressive strength tended to slightly decrease (Liu et al., 2016) [11].

To describe the deterioration and damage in the rock after heat treatment accurately, the damage evolution was presented and applied to the rock under the coupling effect of heat and load (Dougill et al., 1976) [12]. Based on this concept, research on the topic has expanded to include the thermal damage evolution equation, the one-dimensional thermomechanical coupling elasto-brittle damage constitutive equation, and a discussion of the relationship between damage energy release rate and temperature (Liu and Xu, 2000) [13]. Seven new concepts of damage ability and integrity of materials were also introduced; these concepts describe the nature of the two processes of damage and healing, and provide a definition for the concepts of damageability and integrity of materials (Voyiadjis and Kattan, 2017) [14]. A generalized theory of strain equality and a damage constitutive equation of rock under uniaxial compression based on CT testing were presented (Zhang et al., 2003) [15]. The well-known Gurson criterion for materials with radial anisotropy was extended, and the similar influence of “stress softening” was considered (Pensee et al., 2015) [16]. A thermodynamic framework to model inelastic deformation and evolution of anisotropic damage in ductile metals was also presented (Brünig, 2016) [17]. The damage model for the materials subjected to both thermal attack and stress is necessary.

Weibull distribution has been used to establish several thermo-mechanical damage models of rock on the basis of their predecessors, and a new damage model has also been presented (Xu and Karakus, 2018) [18]. Similarly, Weibull distribution has been used to establish the thermomechanical constitutive model, but the compaction stage of rocks in uniaxial compression test has not been reflected well (Xu et al., 2017; Gao et al., 2018) [19,20]. However, it is important to note that granite shows a distinct compaction stage at high temperatures. The compaction stage of rocks in uniaxial compression testing should be considered in the constitutive model. Meanwhile, the constitutive model should reflect the post-failure stage of rock under triaxial compression testing. Therefore, the conventional constitutive model is not suited to these phenomena. In response, when considering the entire process of uniaxial and triaxial compression testing, the compaction stage and the post-failure stage can be reflected in the new TM unified constitutive model by introducing the compaction coefficient *K* and damage variable correction factor *δ* (Liu et al., 2016) [21]. It is expected that this can provide references for studying the mechanical response of the rock damage propagation characteristics in the future.

## 2. Thermomechanical Unified Constitutive Model

### 2.1. Definition of Damage Variable

As a sort of non-independent physical property, the damage must be incorporated into the elastic, plastic, and viscoelastic materials as one type of degradation factor. Therefore, when the damage variable is defined, it should be combined with an independent physical property. Macroscopic damage in materials includes various kinds of defects (such as fragments, flaws, and pores), which can be treated as a kind of continuous medium with a microdamage field. Furthermore, the formation, development, propagation, and accumulation of microdamage are regarded as the process of damage evolution. The damage can be regarded as a part of the microstructure, so it should be introduced into the continuous medium model. Since 1980, many researchers have defined several different damage variables for describing the damage state of a material’s structure (Loland, 1980; Wu and Zhang, 1996) [22,23]; most of them are based on a damage variable, which is defined as the decrease in the effective bearing area of the structure.

As a continuous evolution and non-independent physical property, the damage variable increases when the rock is impacted by an external force, such as uniaxial pressure. Therefore, the Weibull distribution with two parameters is selected to calculate the damage evolution. Since even infinitesimal quantities of rock material obey the Weibull distribution, the Weibull distribution function is shown as:

(1) $$f\left(\varepsilon ;\alpha ,m\right)=\frac{m}{F_{0}}\cdot {\left(\frac{F}{F_{0}}\right)}^{m-1}\cdot e^{-{\left(\frac{F}{F_{0}}\right)}^{m}}$$

The cumulative distribution function is:

(2) $$\int_{0}^{+\infty }f\left(\varepsilon ;\alpha ,m\right)d\varepsilon =1-e^{-{\left(\frac{F}{F_{0}}\right)}^{m}}$$

The damage variable under triaxial compression can be shown as:

(3) $$D=1-e^{-{\left(\frac{F}{F_{0}}\right)}^{m_{0}}}$$

where *F* is the unit with triple-shear Drucker–Prager yield criteria; the formation is shown as:

(4) $$F=\alpha_{0}I_{1}+\sqrt{J_{2}}$$

(5) $$\alpha_{0}=\frac{2sin\varphi }{\sqrt{3}\left(3-sin\varphi \right)}$$

where $F_{0},m_{0}$ are the scale parameter and shape parameter, respectively, both of which are greater than 0. $I_{1},J_{2}$ are the first invariant of the stress tensor and the second invariant of the deviatoric stress tensor, respectively.

Following the Lemaitre theory of strain equality:

(6) $$\left[\sigma^{*}\right]=\frac{\left[\sigma \right]}{1-\left[D\right]}$$

The damage variable correction factor *δ* is added in this formation for controlling and reflecting the post-failure stage of the stress–strain curve under triaxial compression conditions. Therefore, the effective principle equation is:

(7) $$\sigma_{i}{}^{*}=\frac{\sigma_{i}}{1-\delta \left[D\right]},\;i=1,2,3.$$

According to (7), the effective principle is obtained as:

(8) $$\sigma_{1}{}^{*}=\frac{\sigma_{1}}{1-\delta D}$$

(9) $$\sigma_{2}{}^{*}=\sigma_{3}{}^{*}=\frac{\sigma_{2}}{1-\delta D}=\frac{\sigma_{3}}{1-\delta D}$$

Following the generalized Hooke’s law:

(10) $$\varepsilon_{i}=\frac{1}{E}\left[\sigma_{i}{}^{*}-\mu \left(\sigma_{j}{}^{*}+\sigma_{k}{}^{*}\right)\right],\;i,j,k=1,2,3.$$

By substituting (7) into (9), the following relationship is derived:

(11) $$\varepsilon_{i}=\frac{1}{E\left(1-\delta \left[D\right]\right)}\left[\sigma_{i}-\mu \left(\sigma_{j}+\sigma_{k}\right)\right],\;i,j,k=1,2,3.$$

Combining (8), (9), and (11):

(12) $$\varepsilon_{1}=\frac{1}{E\left(1-\delta D\right)}\left[\sigma_{1}-\mu \left(\sigma_{2}+\sigma_{3}\right)\right]$$

As a result of $\sigma_{2}=\sigma_{3}$ in the quasi-triaxial compression test, (12) can be reorganized as:

(13) $$\varepsilon_{1}=\frac{1}{E\left(1-\delta D\right)}\left[\sigma_{1}-2\mu \sigma_{3}\right]$$

Following the effective stress, $I_{1},J_{2}$ can be obtained as:

(14) $$I_{1}=\sigma_{1}{}^{*}+\sigma_{2}{}^{*}+\sigma_{3}{}^{*}=\frac{\sigma_{1}+2\sigma_{3}}{\sigma_{1}-2\mu \sigma_{3}}E\varepsilon_{1}$$

(15) $$\sqrt{J_{2}}=\frac{1}{6}\left[{\left(\sigma_{1}{}^{*}-\sigma_{2}{}^{*}\right)}^{2}+{\left(\sigma_{2}{}^{*}-\sigma_{3}{}^{*}\right)}^{2}+{\left(\sigma_{1}{}^{*}-\sigma_{3}{}^{*}\right)}^{2}\right]=\frac{\left(\sigma_{1}-\sigma_{3}\right)}{\sqrt{3}(\sigma_{1}-2\mu \sigma_{3})}E\varepsilon_{1}$$

In addition, the compaction coefficient *K* is:

(16) $$K=\{\begin{array}{ll} log_{n}\left[\frac{\left(n-1\right)\varepsilon_{1}}{\varepsilon_{c}}+1\right] & \varepsilon <\varepsilon_{c}\\ 1 & \varepsilon \geq \varepsilon_{c} \end{array}$$

By substituting (4) into the stress–strain formula, and combining (13) and (16), the following relationship is derived:

(17) $$\sigma_{1}=E_{0}K\varepsilon_{1}\left[1-\delta +\delta e^{-{\left(\frac{F}{F_{0}}\right)}^{m_{0}}}\right]+2\mu \sigma_{3}$$

According to the boundary condition (i), when $\varepsilon_{1}=\varepsilon_{c}$, $\sigma_{1}=\sigma_{c}$, where $\sigma_{c}$ is the axial peak stress in compression test, $\varepsilon_{c}$ is the strain corresponding to $\sigma_{c}$, $E_{0}$ is the Young’s modulus of rock in the initial state, and $F_{0}$ is the scale parameter of rock in the initial state.

Therefore:

(18) $$\sigma_{c}=E_{0}K_{c}\varepsilon_{c}\left[1-\delta +\delta e^{-{\left(\frac{F}{F_{0}}\right)}^{m_{0}}}\right]+2\mu \sigma_{3}$$

where $K_{c}$ is the value of *K* when the strain arrives at $\varepsilon_{c}$.

The relationship can be derived by (18):

(19) $${\left(\frac{F}{F_{0}}\right)}^{m_{0}}=ln\frac{\delta E_{0}K_{c}\varepsilon_{c}}{\sigma_{c}-2\mu \sigma_{3}+\left(\delta -1\right)E_{0}K_{c}\varepsilon_{c}}$$

According to the boundary condition (ii), when $\varepsilon_{1}=\varepsilon_{c}$, $\frac{d\sigma_{1}}{d\varepsilon_{1}}=0$

(20) $$\frac{d\sigma_{1}}{d\varepsilon_{1}}=E_{0}K\left[1-\delta +\delta e^{-{\left(\frac{F}{F_{0}}\right)}^{m_{0}}}\right]-E_{0}K\varepsilon_{1}\delta e^{-{\left(\frac{F}{F_{0}}\right)}^{m_{0}}}\cdot m_{0}\cdot \frac{F^{m_{0}-1}}{F_{0}{}^{m_{0}}}\cdot \frac{dF}{d\varepsilon_{1}}=0$$

where *F* is:

(21) $$F=\frac{\alpha_{0}\left(\sigma_{1}+2\sigma_{3}\right)+\frac{1}{\sqrt{3}}\left(\sigma_{1}-\sigma_{3}\right)}{\sigma_{1}-2\mu \sigma_{3}}E\varepsilon_{1}$$

Taking the derivative of the implicit function of $\varepsilon_{1}$:

(22) $$\frac{dF}{d\varepsilon_{1}}=E_{0}\left(\frac{1}{\sigma_{1}-2\mu \sigma_{3}}\right)\left[\alpha_{0}\left(\sigma_{1}+2\sigma_{3}\right)+\frac{1}{\sqrt{3}}\left(\sigma_{1}-\sigma_{3}\right)\right]\;-\left(\frac{1}{\sigma_{1}-2\mu \sigma_{3}}\right)\frac{d\sigma_{1}}{d\varepsilon_{1}}\left[\alpha_{0}\left(\sigma_{1}+2\sigma_{3}\right)+\frac{1}{\sqrt{3}}\left(\sigma_{1}-\sigma_{3}\right)\right]E\varepsilon_{1}+\left(\frac{1}{\sigma_{1}-2\mu \sigma_{3}}\right)\left[\alpha_{0}\frac{d\sigma_{1}}{d\varepsilon_{1}}+\frac{1}{\sqrt{3}}\frac{d\sigma_{1}}{d\varepsilon_{1}}\right]E\varepsilon_{1}$$

Boundary conditions (i) and (ii) can be substituted into (22) for the following result:

(23) $$\frac{dF}{d\varepsilon_{1}}{|}_{\sigma_{1}=\sigma_{c}}^{\varepsilon_{1}=\varepsilon_{c}}=\frac{E_{0}\left[\alpha_{0}\left(\sigma_{c}+2\sigma_{3}\right)+\frac{1}{\sqrt{3}}\left(\sigma_{c}-\sigma_{3}\right)\right]}{\sigma_{c}-2\mu \sigma_{3}}$$

where $F_{c}$ is:

(24) $$F_{c}=\frac{\alpha_{0}\left(\sigma_{c}+2\sigma_{3}\right)+\frac{1}{\sqrt{3}}\left(\sigma_{1}-\sigma_{3}\right)}{\sigma_{c}-2\mu \sigma_{3}}E\varepsilon_{c}$$

Combining (22), (23), and (24), the following relationship can be obtained:

(25) $$m_{0}\cdot \frac{F^{m_{0}-1}}{F_{0}{}^{m_{0}}}=\frac{F_{c}\left(\sigma_{c}-2\mu \sigma_{3}\right)}{\left(\frac{dF}{d\varepsilon_{1}}{|}_{\sigma_{1}=\sigma_{c}}^{\varepsilon_{1}=\varepsilon_{c}}\right)\varepsilon_{c}\left[\sigma_{c}-2\mu \sigma_{3}+\left(\delta -1\right)E_{0}K_{c}\varepsilon_{c}\right]}$$

Solving the system of equations:

(26) $$\{\begin{array}{c} {\left(\frac{F}{F_{0}}\right)}^{m_{0}}=ln\frac{\delta E_{0}K_{c}\varepsilon_{c}}{\sigma_{c}-2\mu \sigma_{3}+\left(\delta -1\right)E_{0}K_{c}\varepsilon_{c}}\\ m_{0}\cdot \frac{F^{m_{0}-1}}{F_{0}{}^{m_{0}}}=\frac{F_{c}\left(\sigma_{c}-2\mu \sigma_{3}\right)}{\left(\frac{dF}{d\varepsilon_{1}}{|}_{\sigma_{1}=\sigma_{c}}^{\varepsilon_{1}=\varepsilon_{c}}\right)\varepsilon_{c}\left[\sigma_{c}-2\mu \sigma_{3}+\left(\delta -1\right)E_{0}K_{c}\varepsilon_{c}\right]} \end{array}$$

The parameters $m_{0}$ and $F_{0}$ can be described as:

(27) $$\{\begin{array}{c} m_{0}=\frac{F_{c}\left(\sigma_{c}-2\mu \sigma_{3}\right)}{\left(\frac{dF}{d\varepsilon_{1}}{|}_{\sigma_{1}=\sigma_{c}}^{\varepsilon_{1}=\varepsilon_{c}}\right)\varepsilon_{c}\left[\sigma_{c}-2\mu \sigma_{3}+\left(\delta -1\right)E_{0}K_{c}\varepsilon_{c}\right]ln\frac{\delta E_{0}K_{c}\varepsilon_{c}}{\sigma_{c}-2\mu \sigma_{3}+\left(\delta -1\right)E_{0}K_{c}\varepsilon_{c}}}\\ F_{0}=\frac{F_{c}}{{\left[ln\frac{\delta E_{0}K_{c}\varepsilon_{c}}{\sigma_{c}-2\mu \sigma_{3}+\left(\delta -1\right)E_{0}K_{c}\varepsilon_{c}}\right]}^{\frac{1}{m_{0}}}} \end{array}$$

### 2.2. Definition of Thermal Damage Variable

According to the experimental program, the elastic modulus is selected to define the thermal damage of rock subjected to thermal treatment. After thermal treatment at a certain temperature, the thermal damage of the granite specimen is a constant value. The thermal damage variable $D_{T}$ can be defined as:

(28) $$D_{T}=1-\frac{E_{T}}{E_{0}}$$

where $E_{T}$ is the elastic modulus of granite after thermal treatment at temperature *T*, and $E_{0}$ is the initial elastic modulus of untreated granite.

**Figure 1:**
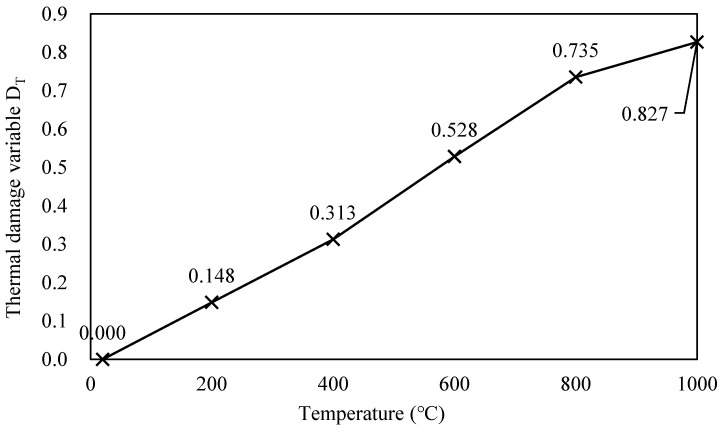
The average values of the damage variables of granite specimens exposed to different temperatures.

The average values of damage variables of granite specimens after thermal treatment at different temperatures are shown in Figure 1. As the temperature increases, the average value of the damage variable increases.

### 2.3. Thermomechanical (TM) Damage Evolution Equation

The total TM damage effect of granite can be expressed by the total damage variable $D_{m}$, which comes from the generalized theory of strain equality. The TM damage variable is defined as:

(29) $$D_{m}=D+D_{T}-DD_{T}$$

The TM unified constitutive model can be described as:

(30) $$\sigma_{1}=\left(1-D_{T}\right)E_{0}K\varepsilon_{1}\left[1-\delta +\delta e^{-{\left(\frac{F}{F_{0}}\right)}^{m_{0}}}\right]+2\mu \sigma_{3}$$

The generalized Hooke’s law is selected to build the TM unified constitutive model. Therefore, the damage of rock caused by high temperature depends entirely on the thermal damage variable $D_{T}$. In a uniaxial compression test, there is no confining pressure around the specimens. Meanwhile, the test specimen is granite, with brittle failure. For these reasons, the axial stress shows a drastic decline after peak stress. In order to ensure that the theoretical peak strain after high-temperature action is close to the experimental peak strain, the peak strain $\varepsilon_{cT}$ obtained by laboratory testing after high temperatures was used instead of the initial peak strain $\varepsilon_{c}$.

Therefore, the scale parameter $F_{T}$ and shape parameter $m_{T}$ in TM unified constitutive model are:

(31) $$\{\begin{array}{c} m_{T}=m_{0}\\ F_{T}=\frac{F_{c}}{{\left[ln\frac{\delta E_{0}K_{c}\varepsilon_{cT}}{\sigma_{c}-2\mu \sigma_{3}+\left(\delta -1\right)E_{0}K_{c}\varepsilon_{cT}}\right]}^{\frac{1}{m_{T}}}} \end{array}$$

**Figure 2:**
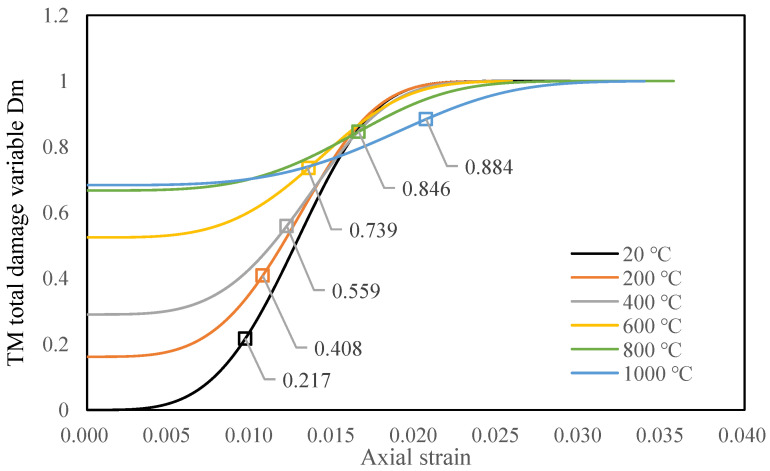
The average values of the damage variables of granite specimens exposed to different temperatures.

**Figure 3:**
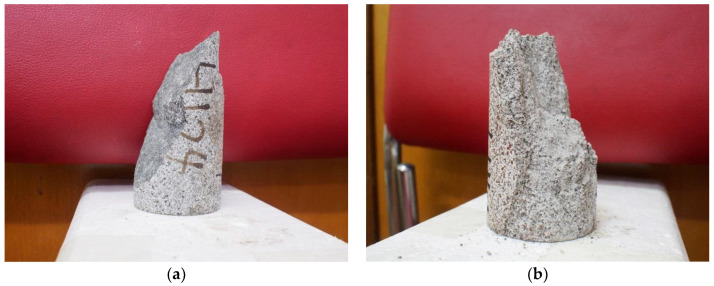
Granite specimens after uniaxial compression tests subjected to heat treatment at (**a**) 200 °C and (**b**) 1000 °C.

Based on (31), the picture of the TM total damage variable $D_{m}$ after heat treatment at different temperatures is shown in Figure 2. It can be observed that the initial damage of granite gradually increases with the increasing temperature; furthermore, the total damage variables at all temperatures gradually approach 1 with the increasing axial strain. Relatively speaking, the TM total damage variables of granites treated at 1000 °C were slightly inconsistent with those treated at other temperatures. From 20 to 600 °C, the total damage variables all granites rose rapidly at a similar strain, and finally approached 1. However, the increasing strain of the TM total damage variable after 800 and 1000 °C treatment showed more distinct changes; meanwhile, the curve became gentler and smoother. From the perspective of data, this phenomenon is caused by the strain softening of granite after exposure to high temperatures. In addition, the theoretical calculation of TM total damage variable curves cannot reflect the failure point of rock specimens directly. Therefore, the TM total damage variable $D_{m}$ only shows the development of microcracks and -pores inside rocks, while the granite specimens have more complicated failure situations. For this reason, the total damage variable $D_{m}$ corresponding to the peak stress during the failure of rock specimens is labeled on the curves of Figure 2. The changes in $D_{m}$ during the failure under the influence of thermal treatment are reflected directly. It can be observed that, with the increase in temperature, the peak strain of granite specimens increases gradually, and the corresponding $D_{m}$ also gradually increases to 1. The $D_{m}$ of the specimens at the failure points increased from 0.217 at 20 °C to 0.844 at 1000 °C, indicating that the granite specimens changed from brittle failure to ductile failure. According to the changes in curve shape, it can be seen that the brittle failure is relatively obvious after 20–600 °C heat treatment, and the specimens break after the $D_{m}$ increases for a short time. After 600 °C heat treatment, the $D_{m}$ curves change shape, and gradually become flat. After 800 and 1000 °C heat treatment, the shape of the $D_{m}$ curves changes greatly, and the values of $D_{m}$ increase gradually. According to the theoretical curve, it can be seen that when the temperature reaches above 800 °C, the properties of granite have changed; therefore, the shapes of damage curves have changed accordingly. Based on the experimental results and theoretical equations, the total damage variable of untreated (20 °C) granite specimens is only 0.217. It can be deduced that the untreated specimens have fewer cracks inside them, and the specimens are relatively intact when they break. The failure surface is complete and dense (Figure 3a). Compared with untreated specimens, the structural damage caused by heat treatment between 600 °C and 1000 °C is comparatively higher. After uniaxial compression tests of granite specimens subjected to 1000 °C, more fragments of the specimens and rock powder are found on the surface of the experiment table, and the failure planes of rock specimens are irregular (Figure 3b).

## 3. Example Verification of the Thermomechanical Damage Evolution Model

**Figure 4:**
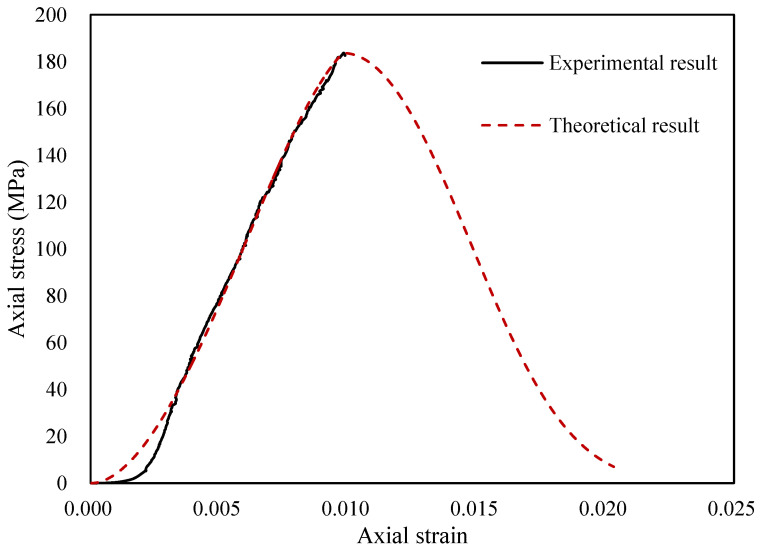
The verification of the TM damage evolution model for an untreated granite specimen.

The stress–strain curve of the uniaxial compression test of granite was selected to verify the theoretical constitutive model. Due to the drastic decrease in the stress after failure under the uniaxial compression test, the machine began fracture protection, and failed to record the full stress–strain curve data. Therefore, in the case of the uniaxial compression test, there was no gentle curve after failure. For this reason, the damage correction coefficient δ equals 1. Figure 4 shows the theoretical stress–strain curve and the evolution curve of the TM-coupled damage evolution model. The theoretical curve is calculated from (29) and the required parameters based on the uniaxial compression test data of granite rock. The parameters of the granite without heat treatment are shown in Table 1. The theoretical curve established by Weibull distribution is in good agreement with the experimental curve of the uniaxial compression test. The uniaxial compressive strength and peak strain obtained from the theoretical model are consistent with the experimental data, and the compaction stage is simulated well.

**Figure 5:**
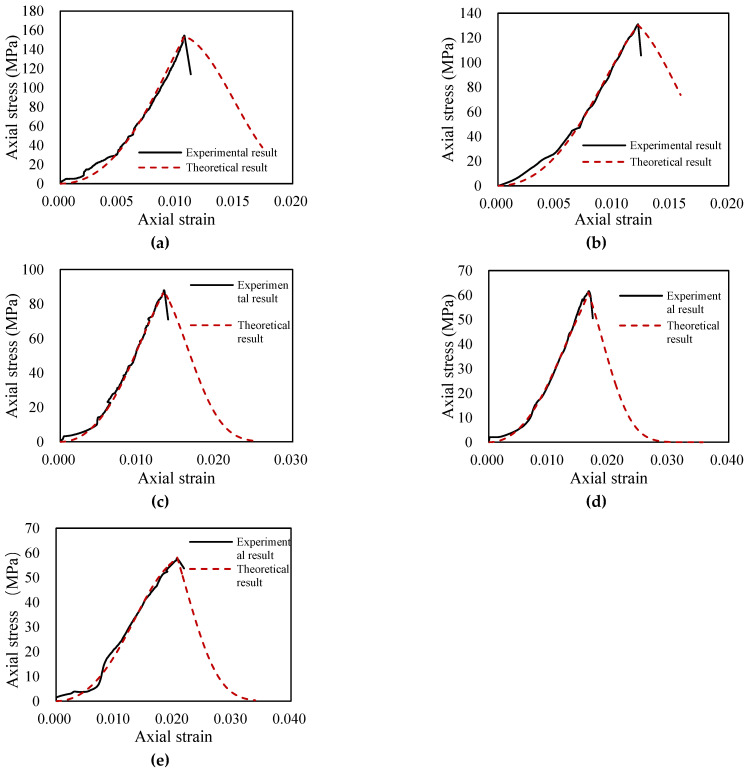
The theoretical and experimental results of uniaxial compression tests of granite subjected to (**a**) 200 °C, (**b**) 400 °C, (**c**) 600 °C, (**d**) 800 °C, and (**e**) 1000 °C.

To verify the damage evolution equation further, the experimental results of different temperatures were compared. Figure 5 shows the theoretical and experimental results of uniaxial compression tests with 200–1000 °C thermal treatments. The parameters used in the stress–strain curve from 200 to 1000 °C are shown in Table 2. When the uniaxial compression test data are used for model validation, the shape parameter $m_{0}$ is taken from the uniaxial compression test without heat treatment. In the uniaxial compression test, the rock specimens are not affected by confining pressure; therefore, the stress–strain curve of the rock always decreases drastically after failure.

Although the stress–strain curves of granite specimens become gentler with increasing temperature, the stress of specimens still decreases drastically when the axial stress reaches the uniaxial compressive strength. For this reason, the damage shape parameters at different temperatures were all taken as $m_{0}$ when uniaxial compression test data were used to verify the three-dimensional model.

The results show that the TM unified constitutive model based on Weibull distribution and triple-shear Drucker–Prager yield criterion, which introduces the compaction coefficient *K* and the damage variable correction factor δ, matches the experimental results accurately.

**Figure 6:**
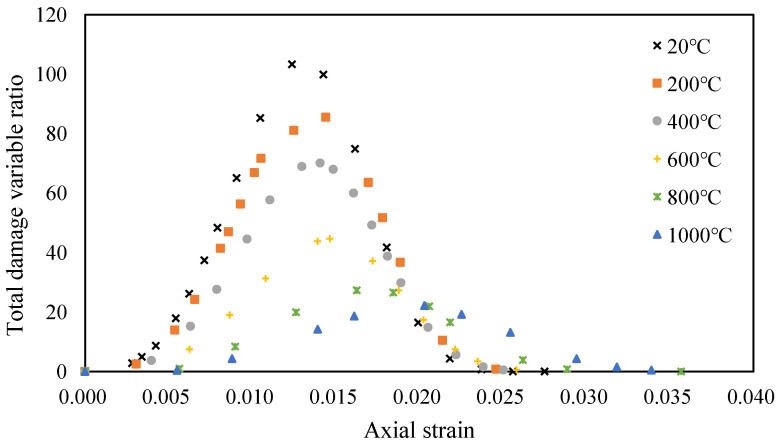
The total damage evolution ratio of granite after heat treatment.

The relationship between the axial strain and the total damage evolution of granite subjected to different temperatures is shown in Figure 6. The results show that the variation trend of the total damage variable ratio has changed. The peak of the total damage variable ratio appears later with increasing temperature. Furthermore, the peak value of the total damage variable ratio also decreases with the increasing temperature. It can be concluded that the granite specimens subjected to higher temperatures will suffer from damage earlier, and that the peak value of the total damage variable ratio will decrease.

## 4. Conclusions

(1) After heat treatment, rock is given a thermal load first, and thermal damage appears inside the rock specimen. Based on this condition, the stress load is the second load coupled with the thermal load. The process is not the mechanical superposition of thermal damage and stress damage but, rather, the coupling effect. Meanwhile, the coupling effect of stress damage and thermal damage is lower compared with the mechanical superposition of them;
(2) The TM unified constitutive model can describe the whole process, including the compaction stage and post-failure stage. The experimental results of uniaxial compression testing are used for verification of the theoretical model. The results show that the theoretical curves match the experimental curves accurately;
(3) The relationship between the total damage evolution ratio and the axial strain of granite subjected to heat treatment at different temperatures can be calculated by the total damage evolution equation. The results show that the peak of the total damage evolution ratio occurs later with increasing temperature. In addition, the peak value of the total damage evolution ratio also decreases with increasing temperature.

## Figures and Tables

**Table 1:** The mechanical parameters of granite without heat treatment.

Initial Young’s Modulus $E_{0}$ (MPa)	Poisson’s Ratio *μ*	Triple-Shear Constant $\alpha_{0}$	Damage Shape Parameter $m_{0}$	Damage Scale Parameter $F_{0}$	Experimental Constant *n*
24.3	0.25	0.52167	3.82	3.72 × 108	3.0

**Table 2:** Model parameters of the TM unified constitutive model at different temperatures, based on Weibull distribution and triple-shear Drucker–Prager yield condition.

Temperature (°C)	200	400	600	800	1000
Initial Young’s Modulus *E*_0_ (MPa)	24.3	24.3	24.3	24.3	24.3
Thermal Young’s Modulus *E_T_* (MPa)	20.4	17.3	11.6	8.09	7.69
Possion’s ratio *m*	0.25	0.25	0.25	0.25	0.25
Triple shear constant *α*_0_	0.52167	0.52167	0.52167	0.52167	0.52167
Damage shape parameter *m*_0_	3.82	3.82	3.82	3.82	3.82
Damage scale parameter *F*_0_	3.72 × 108	3.95 × 108	4.15 × 108	4.73 × 108	5.51 × 108
Experimental constant *n*	0.3	0.3	0.2	0.1	0.11
